# Antimicrobial Drug-resistant *Salmonella* Typhimurium (Reply to Dahl)

**DOI:** 10.3201/eid0910.030029

**Published:** 2003-10

**Authors:** Morten Helms, Pernille Vastrup, Kåre Mølbak

**Affiliations:** * Statens Serum Institut, Copenhagen, Denmark

**In Reply to Dahl:** The emergence and spread of multidrug-resistant *Salmonella* *enterica* serovar Typhimurium DT104 (MDR DT104) contributed to an international increase in antimicrobial drug resistance in *S.* Typhimurium in the late 1990s (*1*,*2*). This type of Salmonella is usually resistant to five drugs: ampicillin, chloramphenicol, streptomycin, sulfonamides, and tetracycline (R-type ACSSuT) and easily acquires resistance to other drugs, including quinolones, trimethoprim, and aminoglycosides (*1*,*3*–*5*). To determine death rates after infection with MDR DT104 or closely related strains, we identified patients who were infected with strains at least resistant to ACSSuT (*6*). Analysis limited to strains that were only R-type ACSSuT would have given a misleading result since MDR DT104 often, as mentioned, develops additional resistance to other classes of antimicrobial drugs in addition to the ACSSuT-complex. This fact needs to be taken into account in any attempt to quantify the overall public health impact of MDR DT104 and related strains.

We found, in our matched cohort study (*6*), that 283 patients infected with strains resistant to at least ACSSuT were 4.8 times more likely to die than the general Danish population, compared with 2.3 for 953 patients infected with pansusceptible strains. This difference in death rates ocurred mainly because 40 of the 283 strains had R-type ACSSuTNx (i.e., additional resistance to nalidixic acid), and infection with this strain in particular is associated with a high death rate (relative mortality 13.1). As Dahl suggests, infection with R-type ACSSuT (Nx susceptible) was not associated with excess mortality in the 243 patients included in the analysis, and the measured effect of ACSSuT was achieved by the inclusion of the nalidixic acid–resistant strains in this group. However, all deaths associated with nalidixic acid–resistant strains occurred in the 40 patients with R-type ACSSuTNx (being DT104s), whereas none of the 43 patients infected with non-ACSSut strains resistant to nalidixic acid died. This finding may be related to small numbers in these subanalyses. However, because 25 of the patients with R-type ACSSuTNx were part of an outbreak, they may have had an average higher exposure dose, which may have contributed to some deaths (*3*). In addition, an interaction between different resistance traits in Salmonella may exist, which may lead to more deaths and disease, or DT104 may be somewhat more virulent than most other *S.* Typhimurium subtypes.

The database that we used for our analysis was updated in May 2002. We have now identified 13 deaths in 342 patients infected with strains resistant to ACSSuT (but Nx susceptible), which corresponds to a relative mortality rate of 4.18 (95% confidence interval [CI]) 2.18 to 8.02) compared with a matched sample of the general population. Of 1,432 patients infected with pansusceptible strains, 43 patients died (relative mortality rate 2.64; 95% CI 1.88 to 3.70). In other words, the mortality rate in patients infected with strains resistant to ACSSuT (Nx susceptible) was 1.6 times higher than in patients with pansusceptible strains (p value for homogeneity 0.22). These estimates were not adjusted for coexisting conditions as were the estimates in the paper (*6*).

We agree with Dahl that particular problems are associated with quinolone resistance in zoonotic salmonellae and that fluoroquinolones may have reduced efficacy to treat patients infected with *Salmonella* strains that are nalidixic acid (quinolone) resistant (*7*). We therefore encourage initiatives to preserve the efficacy of fluoroquinolones, including a limitation of their use in agriculture. Whether infection with *S.* Typhimurium R-type ACSSuT, with no additional resistance, is associated with higher disease or death rates than pansusceptible *S.* Typhimurium remains unclear. Although the difference was not significant (p=0.22), our recent estimates suggest that the death rate is approximately 60% higher in patients infected with such strains. This view is corroborated by recent studies from the United States, which suggest that *S.* Typhimurium R-type ACSSuT is associated with an increased risk for blood stream infection (*8*) and that resistance in nontyphoid *Salmonella* is associated with an increased risk for admission to hospital (*9*).
